# Nematodes Go Viral

**DOI:** 10.1371/journal.pbio.1001011

**Published:** 2011-01-25

**Authors:** Robin Meadows

**Affiliations:** Freelance Science Writer, Fairfield, California, United States of America

**Figure pbio-1001011-g001:**
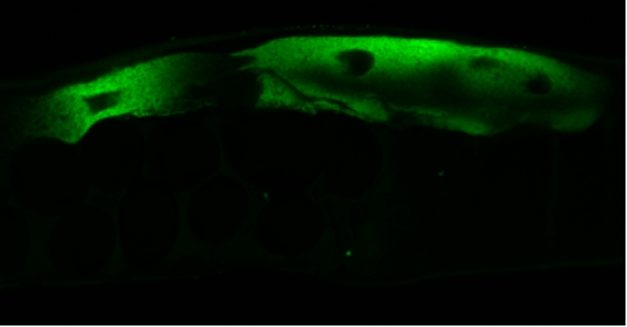
FISH staining demonstrating infection of intestinal epithelial cell of *C. elegans* by Orsay virus.

[Fig pbio-1001011-g001]Tiny worms with simple genetics, nematodes have almost everything it takes to be a favored model system to study viral infections. Recent work has shown that the nematode *Caenorhabditis elegans*, long known to enlist innate immune pathways against bacteria, can also muster such resistance to viruses. And because these immune responses function much the same way in vertebrates, the worm could help answer questions about the role of these pathways in other species, including humans. So what's missing?

Because no viruses were known to infect nematodes naturally, biologists had to rely on artificial means of introducing viruses or viral RNA. But these studies had the drawback of assessing immunity only to viral genome replication. Thus, the question of how the immune system guards against other stages of the virus life cycle, from cell entry to egress, has remained a mystery. Now, biologists may finally get a more comprehensive look at viral infection and immunity in nematodes. In this issue of *PLoS Biology*, Marie-Anne Félix, Eric Miska, David Wang, and colleagues report the first viruses known to infect nematodes naturally in the wild.

The researchers started by collecting nematodes from rotting fruit in French orchards and vineyards. Light microscopy revealed that the worms had striking intestinal symptoms, including cells with degenerated nuclei and cell fusion, suggesting that they were infected. As is true for other animals, the intestine is exposed to ingested microbes and is a main entry point for pathogens in nematodes. However, there were no obvious pathogens, suggesting that the worms might be infected by viruses, which are too small to be seen with light microscopes. Surprisingly, despite the severity of their affliction, the worms led rather normal lives and kept moving, eating, and reproducing, albeit at lower levels.

The researchers set off on their virus hunt by investigating two of the afflicted strains: a *C. elegans* strain from a rotting apple in Orsay and a *Caenorhabditis briggsae* strain from a snail on a rotting grape in Santeuil. Multiple lines of evidence suggested that their intestinal symptoms were viral. For example, afflicted nematodes were cured by bleaching and then reinfected when mixed with filtrates of symptomatic worms. Moreover, electron microscopy revealed virus-sized particles in intestinal cells of symptomatic nematodes but not in those that had been bleach-cured, bolstering the hypothesis that the affliction was viral.

Molecular analysis then indicated that each of the two nematode strains contained RNA sequences that were similar to those of nodaviruses, a family of viruses that infect invertebrates and vertebrates. Based on phylogenetic analysis of the proteins predicted from these RNA sequences, the researchers concluded that the sets of sequences comprised two novel viruses, one from each strain, that are distantly related to known nodaviruses and most closely related to each other. These new viruses were named for the places where they were found, with the “Orsay” virus infecting only *C. elegans* and the “Santeuil” virus infecting only *C. briggsae*.

Previous studies of nematode immunity to artificially introduced viral RNA had shown that nematodes counter infection via the RNA interference (RNAi) pathway, which suppresses replication of viral genomes by destroying RNA sequences. This is a key defense mechanism against RNA viruses common to plants and animals. To see if this also holds for natural viral infections, the researchers determined whether Orsay virus–infected *C. elegans* produced the small RNAs—the short non-coding sequences that mediate and are products of the RNAi pathway. Of the nearly 1.5 million unique small RNAs identified in the infected *C. elegans* strain, about 21,000, or nearly 2%, mapped to the Orsay virus RNA. After bleach-curing, however, the strain no longer produced this subset of small RNAs. These findings suggest that, in keeping with the earlier works that used artificially introduced viral RNA, nematodes do indeed fight naturally occurring viral infections with RNAi.

This conclusion was further strengthened by the finding that RNAi pathway mutations affected viral replication in another *C. elegans* strain. Called N2, this strain can also be infected by the Orsay virus but at far lower levels and with no discernable intestinal symptoms. However, the researchers found that both viral RNA levels and intestinal symptoms were bumped up in N2 worms that harbor a mutant *rde-1* gene, which codes for a protein required for activation of the RNAi pathway. Likewise, Orsay virus–infected N2 worms that had mutations in other RNAi pathway genes, including *rde-2*, *rde-4*, and *mut-7*, also had higher levels of viral RNA. This work opens the way to using Orsay virus–infected mutants to identify additional genes that are instrumental in this mode of anti-viral immunity in nematodes.

The researchers have also found evidence suggestive of other anti-viral defenses in nematodes. Comparison of wild *C. elegans* collected from around the world showed that even though some of the strains had defective RNAi pathways, they still resisted Orsay virus infection. This means the infected strains could be used to identify additional anti-viral pathways in nematodes.

The discovery of virus-infected nematodes in nature proves the worm's value as a model system for studying viral infection. In addition to enabling exploration of defenses against viruses at all stages of their life cycle, the worm will help researchers understand how anti-viral immunity evolves as well as how hosts and pathogens coevolve with each other. And with a wealth of genetic tools already in place, this tiny, transparent model organism will surely help uncover more mechanisms of innate immunity. All eyes in the world of molecular immunology will be watching to see what discoveries this system yields next.

**Félix M-A, Ashe A, Piffaretti J, Wu G, Nuez I, et al. (2011) Natural and Experimental Infection of** ***Caenorhabditis*** **Nematodes by Novel Viruses Related to Nodaviruses. doi:10.1371/journal.pbio.1000586**

